# Carbon Allocation into Different Fine-Root Classes of Young *Abies alba* Trees Is Affected More by Phenology than by Simulated Browsing

**DOI:** 10.1371/journal.pone.0154687

**Published:** 2016-04-28

**Authors:** Tina Endrulat, Nina Buchmann, Ivano Brunner

**Affiliations:** 1 Forest Soils and Biogeochemistry, Swiss Federal Institute for Forest, Snow and Landscape Research WSL, Birmensdorf, Switzerland; 2 Institute of Agricultural Sciences, Swiss Federal Institute of Technology ETH, Zürich, Switzerland; University of Nottingham, UNITED KINGDOM

## Abstract

*Abies alba* (European silver fir) was used to investigate possible effects of simulated browsing on C allocation belowground by ^13^CO_2_ pulse-labelling at spring, summer or autumn, and by harvesting the trees at the same time point of the labelling or at a later season for biomass and for ^13^C-allocation into the fine-root system. Before budburst in spring, the leader shoots and 50% of all lateral shoots of half of the investigated 5-year old *Abies alba* saplings were clipped to simulate browsing. At harvest, different fine-root classes were separated, and starch as an important storage compartment was analysed for concentrations. The phenology had a strong effect on the allocation of the ^13^C-label from shoots to roots. In spring, shoots did not supply the fine-roots with high amounts of the ^13^C-label, because the fine-roots contained less than 1% of the applied ^13^C. In summer and autumn, however, shoots allocated relatively high amounts of the ^13^C-label to the fine roots. The incorporation of the ^13^C-label as structural C or as starch into the roots is strongly dependent on the root type and the root diameter. In newly formed fine roots, 3–5% of the applied ^13^C was incorporated, whereas 1–3% in the ≤0.5 mm root class and 1–1.5% in the >0.5–1.0 mm root class were recorded. Highest ^13^C-enrichment in the starch was recorded in the newly formed fine roots in autumn. The clipping treatment had a significant positive effect on the amount of allocated ^13^C-label to the fine roots after the spring labelling, with high relative ^13^C-contents observed in the ≤0.5 mm and the >0.5–1.0 mm fine-root classes of clipped trees. No effects of the clipping were observed after summer and autumn labelling in the ^13^C-allocation patterns. Overall, our data imply that the season of C assimilation and, thus, the phenology of trees is the main determinant of the C allocation from shoots to roots and is clearly more important than browsing.

## Introduction

European silver fir (*Abies alba* Mill.), a typical tree of montane and lower subalpine forests in the Alps, has a highly relevant protective function against landslide due to its deep rooting system [1], its high resistance against bark beetles (*Ips typographus* L.), and its general ease of natural regeneration [2]. It produces high quality wood that is used for furniture, construction timber and pulp. However, *A*. *alba* has been declining throughout its natural range since the 18^th^ century due to forest management practices [2] and anthropogenic air pollution. Moreover, the high population number of game animals, e.g. deer, and, therefore, high grazing pressure on young *A*. *alba* trees is thought to prevent regeneration [3,4], especially due to browsing of the leader shoot, reducing *A*. *alba* height growth for several years [5].

Although stress responses of different conifers to either artificial or natural defoliation through clipping or herbivory have been studied extensively [6–10], information on belowground responses is scarce. Based on studies with non-woody plants, it has been suggested that herbivore-mediated shifts in belowground carbon (C) allocation patterns of plants are the result of a close coupling of above- and belowground processes in terrestrial ecosystems, induced by plant defense mechanisms [11,12]. The first studies on belowground responses of trees focussed on root growth, either directly influenced by mammalian browsing of shoots or indirectly by mammalian trampling [13,14]. Hester et al. [9] were the first studying the effect of simulated browsing on above- and belowground growth of different tree species in a pot-experiment and reported not only negative above- but also negative belowground responses due to simulated browsing by clipping. Recent studies also addressed the fate of assimilated C belowground and the belowground starch storage responses to browsing events [15–20], with only one study so far applying isotopically labelled C [21]. Although the ^13^C-isotope label in that latter study was measured only in the tree rings of the stems without observing any significant effects of the browsing, this study showed the potential of the ^13^C-isotope application for tracking recently fixed photoassimilates within the plants. Moreover, the ^13^C-isotope method provides the location of ^13^C-incorporation (e.g. root tips, coarse roots) as well as the form of the incorporated ^13^C-label (e.g. starch, cellulose) [22].

The main objectives of the present study were to investigate the effects of browsing, simulated through clipping, on the C allocation belowground at different times during the growing season, to distinguish C incorporation among different types of fine roots, and to investigate the changes in starch storage patterns in the fine roots due to clipping. Deduced from these objectives we tested the following hypotheses: (1) the time point (seasons) of the ^13^C-pulse-labelling and, thus, phenology has a strong effect on the allocation of the ^13^C-label from the shoot to the roots, (2) the incorporation of the ^13^C-label as structural C or as starch into the roots is strongly dependent on the root type and the root diameter, and (3) clipping strongly influences the amount of allocated ^13^C-label as well as the site of incorporation and the form of the incorporated ^13^C-label (structural C *versus* starch) within the root system. Therefore, 5-year-old *A*. *alba* saplings, both clipped and control trees, were pulse-labelled with ^13^CO_2_, either in spring, summer, or autumn, and the assimilated ^13^C was traced into the fine-root systems, with specific emphasis on the incorporation of ^13^C into starch as the main storage carbohydrate.

## Material and Methods

### Experimental design and plant material

The experiment was carried out in 2008 in the tree nursery of the Swiss Federal Research Institute (WSL) in the Swiss lowlands (Birmensdorf, 47°22 N, 08°27 E, 550 m a.s.l.). According to Palacio et al. [15], saplings of an approximate height of 30–50 cm height are considered to be one of the most vulnerable stages of a young tree in relation to large herbivore browsing (compare also [6,9]). Thus, in total, 68 five-year-old nursery-grown *A*. *alba* saplings (height ±30 cm; provenance Beggingen, 47°47 N, 08°33 E, 650 m a.s.l.) were grown in a nursery soil with a pH of 7.2 and were fertilised once at the age of four years with organic NPK fertiliser (Unikorn II, Hauert, Switzerland; 20 g m^–2^).

In the beginning of April 2008, the tree saplings were excavated with their root systems attached, using a root-cutting machine ('Beetroder', Baertschi Agrartechnik AG, Hüswil, Switzerland). The saplings were then replanted into pots (22.5 cm height, 26 cm in diameter) and assigned to two treatments, 35 plants in a browsing treatment and 33 plants in an untreated control. The browsing treatment was performed at the end of April, just prior to bud burst, when a maximum of browsing by wild ungulates can be assumed [23]. The browsing was carried out according to Häsler et al. [10], with clipping two-thirds of the previous year’s leader shoots and one-third of the previous year’s shoot growth at every second lateral branch (proportion of buds removed: 56±1.3%), using diagonal pliers to break the plant tissue in a comparable way to ungulate browsing.

All saplings were randomly arranged in four rows of 17 trees and shaded with wooden frames above the trees and shading nets at the sides. Thus, the trees received only about one-third of direct sunlight, simulating conditions as in the forest understory [10].

### ^13^CO_2_ pulse-labelling

The ^13^CO_2_ pulse-labelling was conducted with three randomly assembled groups of *A*. *alba* tree individuals. Each group was labelled during one physiologically significant phase, i.e., in spring (early May 2008, subsequent to sprouting; 28 trees), in summer (early July 2008, period of stem growth; 20 trees) and in autumn (end of September 2008, end of the growing season; 12 trees). In total, 60 trees were labelled with ^13^CO_2_. In addition to these 60 trees, eight trees (four clipped and four controls) remained unlabelled and were used to calculate the respective ^13^C enrichment in the trees (Table 1).

**Table 1:** Number of harvested and ^13^CO_2_ pulse-labelled trees during the seasons in two years for two clipping treatments and three ^13^CO_2_ labelling seasons. Treatments are 'Control' = unclipped trees, and 'Clipped' = clipped trees.

Treatment	^13^CO_2_ labelling season	Harvesting season				Sum of trees
		Spring	Summer	Autumn	Spring+1 yr	
Control	Spring	4	4	4	1	13
	Summer	-	4	4	2	10
	Autumn	-	-	4	2	6
	Sum of trees	4	8	12	5	29
Clipped	Spring	4	4	4	3	15
	Summer	-	4	4	2	10
	Autumn	-	-	4	2	6
	Sum of trees	4	8	12	7	31

At each labelling date, randomly chosen groups of four *A*. *alba* trees were lined up side by side, and the shoots of the four trees (two clipped and two control trees) were covered with one polyethylene bag (volume approx. 300 l, 100 μm thickness) and pulse-labelled with ^13^CO_2_. The polyethylene bags were then tightly wrapped at the stems, below the crowns, and sealed with string and adhesive tape. With a syringe, 480 ml of gaseous ^13^CO_2_ (CIL Cambridge Isotope Laboratories, Switzerland; 280 mg ^13^C) were added to each polyethylene bag. This equalled 120 ml ^13^CO_2_ (70 mg ^13^C) for each tree individual. In spring, 7 polyethylen bags with total 28 trees were labelled, in summer 5 bags with 20 trees, and in autumn 3 bags with 12 trees.

The addition of the gas was performed following Kagawa et al. [24] and Endrulat et al. [22] to ensure efficient incorporation of the label. After closure of the bags, 2 x 40 ml ^13^CO_2_ were injected into each bag, followed by another 2 x 40 ml after 15 minutes. The trees were left in the bags for 1 h, before bags were opened for another hour. This procedure was repeated three times within one day. The labelling always started around 11 a.m. and lasted until 4 p.m. (summertime) in the afternoon. Since we aimed that plants were exposed to full sunlight for all three labelling dates, we started close to noon because in autumn fog prevails in the mornings. To reduce water condensation and thus limitation of CO_2_ incorporation rate, two electric fans per bag were used to ensure continuous circulation within the bag. The fans were equipped with 80 g silica gel in the front to additionally reduce humidity in the bags. Maximum temperatures at the labelling dates were 21.3°C (May), 23.8°C (July) and 14.5°C (September).

### Tree measurements

Before and after the clipping treatment and immediately before each sapling was harvested, non-destructive morphological measurements were carried out for each tree. These measurements included sapling height, length of new leader shoot, number of buds, number of new shoots (terminals and laterals), and number of branches off the main stem. Furthermore, the origin of the new leader was recorded as originating from an apical, interwhorl or secondary whorl bud according to Häsler et al. [10] (S1 Table).

### Sampling and harvest

Immediately after each of the last labelling exposures, 10 needles of every labelled tree were randomly sampled, milled and analyzed for the maximum ^13^C label in each tree. The maximum ^13^C contents in the needles were then used to calculate the relative ^13^C contents in the roots. Whole trees were harvested at one to four different time points after the three ^13^C-labelling dates: In spring, summer, and autumn 2008, and as well as in spring 2009, i.e., 1, 3, 5, and 12 months after a labelling event (Table 1). Of each harvest, four replicate saplings of both treatments (clipped and control) were harvested, except in the spring 2009, one year after the start (spring+1 yr), when a maximum of three replicates was harvested. At each harvest, two non-labelled control trees were sampled as well.

The whole root systems were carefully washed and partitioned into various root categories using a vernier caliper according to Endrulat et al. [22] to keep track of the spatial distribution of the ^13^C-label within the roots; new roots (newly formed white-coloured fine roots), fine roots of various size classes (≤0.5 mm in diameter, >0.5–1 mm in diameter, >1.0–1.5 in diameter mm, >1.5–2 mm in diameter; brown-coloured), and coarse roots (>2 mm in diameter; brown-coloured).

Branches and needles as well as stems were weighed (fresh weight), frozen in liquid nitrogen, and then stored at -20°C until lyophilisation and biomass (dry weight) determination.

### Starch extraction

Starch was extracted from two fine-root classes, from the newly formed fine roots and the fine roots with a diameter ≤0.5 mm, and in spring, summer, and autumn of 2008. Starch extraction followed a protocol of Smith and Zeeman [25], modified according to Regier et al. [26] and Endrulat et al. [22]. Approximately 60 mg (dry weight; DW) of lyophilized and milled *A*. *alba* fine roots were extracted with 80% ethanol, and boiled to gelatinise the starch. Starch was then digested with α-amylase and amyloglucosidase (Roche, Switzerland) in water solution. After digestion, the starch amount was measured as released glucose with a spectrophotometer (Tecan, Switzerland) at 340 nm. For ^13^C isotopic measurements, the samples were filtrated with centrifugal Filter Devices (Microcon YM-10; Millipore, Switzerland) in order to take out the enzymes according to Göttlicher et al. [27], and then dried in tin capsules (Säntis Analytical AG, Switzerland) in a vacuum centrifuge (Heto Lab Equipment, Denmark).

### δ^13^C analyses and isotope calculations

Carbon isotope and total C concentration analyses in needles, bulk fine-roots and starch were carried out with an Elemental Analyser (EA-1110, Carlo Erba, Italy; Euro EA 3000, Hekatech, Germany) connected to a continuous flow mass spectrometer (Delta V Advantage, Thermo Fisher Scientific, Germany) at WSL. The measured isotope ratios are expressed in δ-notation in permil units (‰), using Vienna-PeeDee Belemnite (V-PDB) as standard. The precision for ^13^C analysis was ±0.2‰.

The amounts of assimilated ^13^C were calculated according to Philip and Simard [28] and Keel et al. [29]. δ^13^C (‰) and C (%) of labelled samples and unlabelled controls were used to calculate the ^13^C enrichment as described in Endrulat et al. [22]. The needle δ^13^C values immediately after each last pulse-labelling exposure were used as maximum signal of each tree individual according to Keel et al. [29]. The ^13^C enrichment (mg) of the needles was set to 100%, and the measured ^13^C contents in all fine-root samples were expressed in percentage of the maximum needle signals, and referred to as relative ^13^C content (%). This approach enables the comparison of all tree individuals due to the elimination of the signal strength variation caused by variable CO_2_ uptake during the labelling.

For ^13^C analyses in roots, three fine-root classes were analysed: newly formed fine roots, fine roots of ≤0.5 mm in diameter, and fine roots of >0.5–1 mm in diameter. For ^13^C labelled starch of fine roots, only the newly formed fine roots and the fine roots of ≤0.5 mm in diameter were analysed.

### Statistical analyses

All statistical analyses were performed using STATVIEW 5.0 (SAS Institute Inc., Cary, NC, USA). The effects of the clipping treatment and the various harvesting seasons on plants and root properties were analysed by two-way analyses of variance (ANOVA) with using Fisher’s protected least significant difference (PLSD). The data were tested for normal distribution and homogeneity of variance. Starch and relative ^13^C content data were log-transformed in order to meet the requested criteria. One data set was not normally distributed, therefore, the non-parametric Mann-Whitney (for two groups) and Kruskal-Wallis tests (for more than two groups) were applied.

## Results

### Biomass partitioning

The clipping of the trees caused over the whole investigation period a significant decrease of aboveground biomass (-5.8%) as well as of the needles (-13.8%; Tables 2 and 3). Subsequently, this loss resulted in a significant increase of the percentage of belowground biomass (+11.9%; Table 3), as well as in a significant increase of the proportion of biomass in coarse (+8.6%) and fine roots (+16%) (Tables 2 and 3). The root/shoot ratio of clipped trees remained more or less on a constant level (0.56–0.63), whereas the ratio of control trees at the beginning in spring was low (0.41) and increased constantly until autumn (to 0.60; Table 2).

**Table 2:** Total biomass (g±SE), root/shoot ratio, and percentage of the various tree components of control (unclipped) and clipped trees for two clipping treatments and four harvesting seasons. Aboveground is the sum of stem, branches, and needles, belowground is the sum of coarse and fine roots. Different letters indicate significant differences among harvesting seasons within one treatment (*P*<0.05). * *P*<0.05, ** *P*<0.01, and *** *P*<0.001 indicate significant differences between 'Control' and 'Clipped' trees.

Treatment	Harvesting season	Trees (n)	Total biomass (g; ±SE)	Root / shoot ratio	Tree components (%)						
					Stem	Branches	Needles	Coarse roots	Fine roots	Above-ground	Below-ground
Control	Spring	5	48.9±7.0^c^	0.41^b^	17.3	14.2	39.4	17.2	11.9	70.9	29.1
	Summer	9	70.5±6.4^bc^	0.44^b^	18.7	14.5	36.5	18.8	11.4	69.8	30.2
	Autumn	13	81.6±6.4^b^	0.60^a^	16.4	14.4	31.6	19.5	18.0	62.5	37.5
	Spring+1 yr	6	104.8±10.5^a^	0.54^a^	13.4	11.8	40.3	18.2	16.3	65.5	34.5
	Mean		77.8±4.6	0.52	16.6	13.9	35.7	18.8	15.0	66.3	33.7
Clipped	Spring	5	41.2±8.5^b^	0.59	18.1	12.4	32.8**	19.7	16.9	63.4*	36.6*
	Summer	9	59.2±9.3^ab^	0.57***	21.4*	14.2	28.4***	20.8	15.3*	64.0**	36.0**
	Autumn	13	72.6±4.8^a^	0.63	18.4*	15.6	27.7***	22.2*	16.2	61.7	38.4
	Spring+1 yr	8	67.6±8.4^a^*	0.56	14.2	10.7	39.2	17.6	18.3	64.1	35.9
	Mean		63.5±4.0	0.59	18.2	13.7	31.2	20.0	16.5	63.1	36.9

**Table 3:** Results of two-way ANOVA testing the effect of the clipping treatment (C) and the harvesting seasons (H) (spring, summer, autumn, spring+1 yr) on total biomass and root/shoot ratio as well as on the percentage of the various tree components. Significant effects (*P*<0.05) are given in bold.

Tree components	Clipping (C)			Harvesting season (H)			C x H		
	*df*	*F*	*P*	df	*F*	*P*	*df*	*F*	*P*
Total biomass	1	8.31	**0.006**	3	8.02	**<0.001**	3	1.47	0.231
Root/shoot ratio	1	14.57	**<0.001**	3	7.24	**<0.001**	3	2.78	**0.049**
Stem	1	5.34	**0.024**	3	15.09	**<0.001**	3	0.48	0.698
Branches	1	0.75	0.391	3	9.52	**<0.001**	3	1.45	0.237
Needles	1	47.35	**<0.001**	3	43.28	**<0.001**	3	4.76	**0.005**
Coarse roots	1	4.40	**0.040**	3	3.54	**0.020**	3	1.02	0.390
Fine roots	1	5.93	**0.018**	3	5.24	**0.003**	3	3.31	**0.026**
Aboveground	1	17.02	**<0.001**	3	8.28	**<0.001**	3	3.15	**0.031**
Belowground	1	17.02	**<0.001**	3	8.28	**<0.001**	3	3.15	**0.031**

Both, clipping treatment and harvesting season, influenced the tree parameters significantly, with the one exception of the branches after the clipping treatment (Table 3). Interactions of the two factors, clipping and harvesting, occurred for the root/shoot ratio, the above- and belowground biomass, the needles, and the fine roots (Table 3). This fact indicates that the effects of clipping upon these tree parameters depend on the harvesting season.

**Fig 1:**
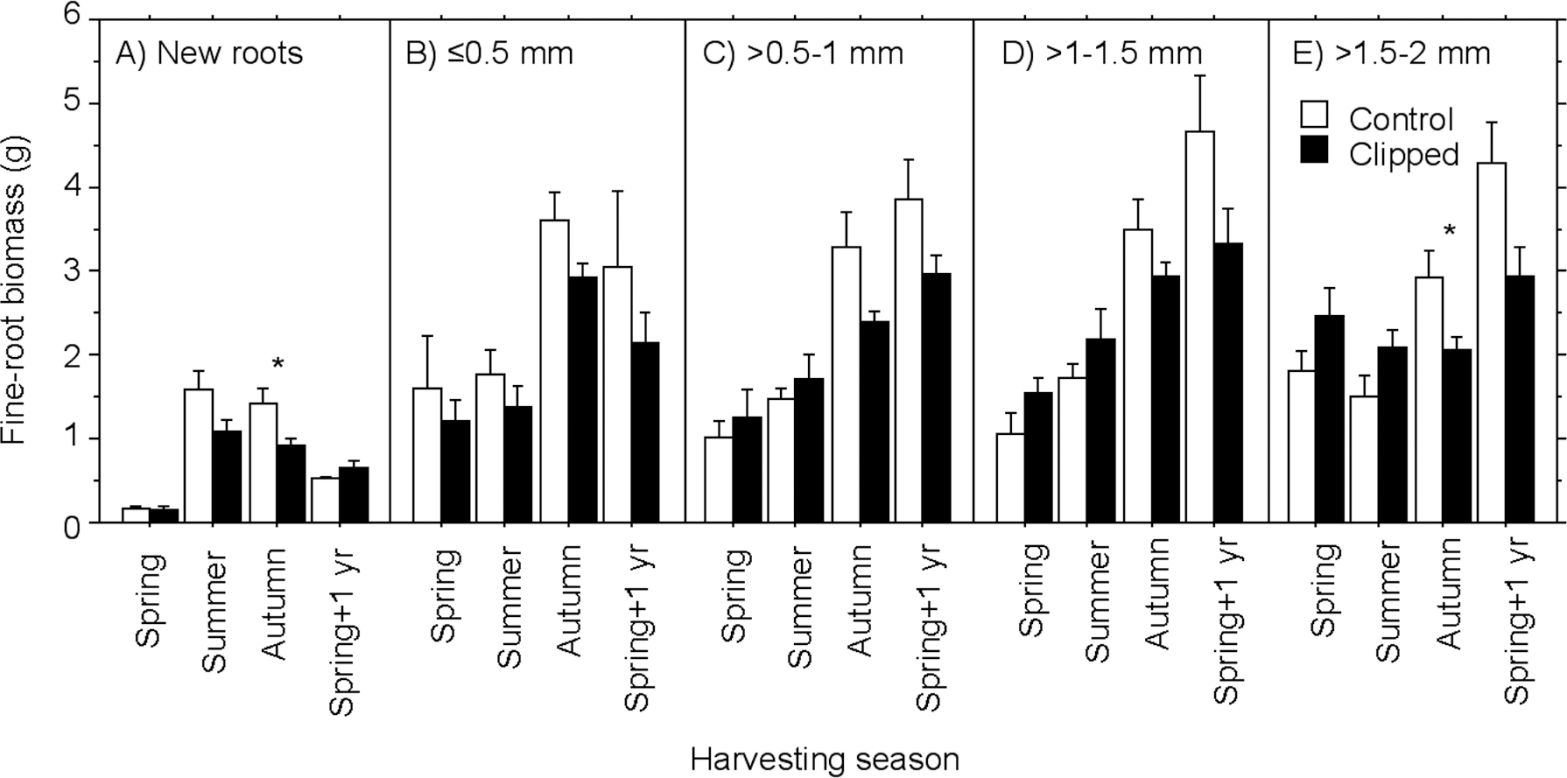
Mean biomass (g, ±SE; n = 5–13, see S1 Table) of fine-root classes of control and clipped trees: (A) new roots, (B) ≤0.5 mm, (C) >0.5–1.0 mm, (D) >1.0–1.5 mm, (E) >1.5–2.0 mm. Significant differences between control and clipped trees are given with * (*P*<0.05).

Separating the fine roots into newly formed roots and into different diameter classes (Fig 1), we found that root biomass differed among various fine-root classes in all harvesting seasons with the exception of summer (Table 4). These significant effects were mainly caused by the small biomass of the newly formed fine roots (Fig 1). The clipping treatment caused a significant reduction of fine-root biomass in autumn and spring+1 yr (Table 4, Fig 1), but not in spring or summer (Table 4).

**Table 4:** Results of two-way ANOVA testing the effect of the clipping treatment (C) and the various fine-root classes (R; new roots, size classes ≤0.5 mm, >0.5–1.0 mm, >1.0–1.5 mm, and >1.5–2.0 mm) on fine-root biomass at various harvesting seasons. Significant effects (*P*<0.05) are given in bold (autumn data were tested with non-parametric tests due to non-normal distribution).

Harvesting season	Clipping (C)			Fine-root classes (R)			C x R		
	*df*	*F*	*P*	df	*F*	*P*	*df*	*F*	*P*
Spring	1	1.00	0.324	4	11.41	**<0.001**	4	0.98	0.429
Summer	1	0.26	0.609	4	1.90	0.1181	4	2.14	0.083
Autumn	1	-	**0.007**	4	-	**<0.001**	-	-	-
Spring+1 yr	1	9.77	**0.003**	4	19.10	**<0.001**	4	0.94	0.446

### ^13^C in needles and fine-roots

The mean recovery of the ^13^C isotope (ratio of incorporated ^13^C_needles_ to the amount of ^13^C added to one tree) after the pulse-labelling was 71% (±8.2%) for control and 55% (±6.2%) for clipped trees. However, mean δ^13^C values in needles directly after the last labelling exposure varied significantly among trees of the same labelling bag (*P*≤0.019 for all bags) and among the three labelling dates (*P*<0.001). Clipped and unclipped control trees did not differ significantly in their foliar δ^13^C values (*P =* 0.391), and no significant interaction between treatment and labelling date was found (*P* = 0.078), indicating similar conditions for all labelling dates and no difference in uptake between the two treatments.

**Fig 2:**
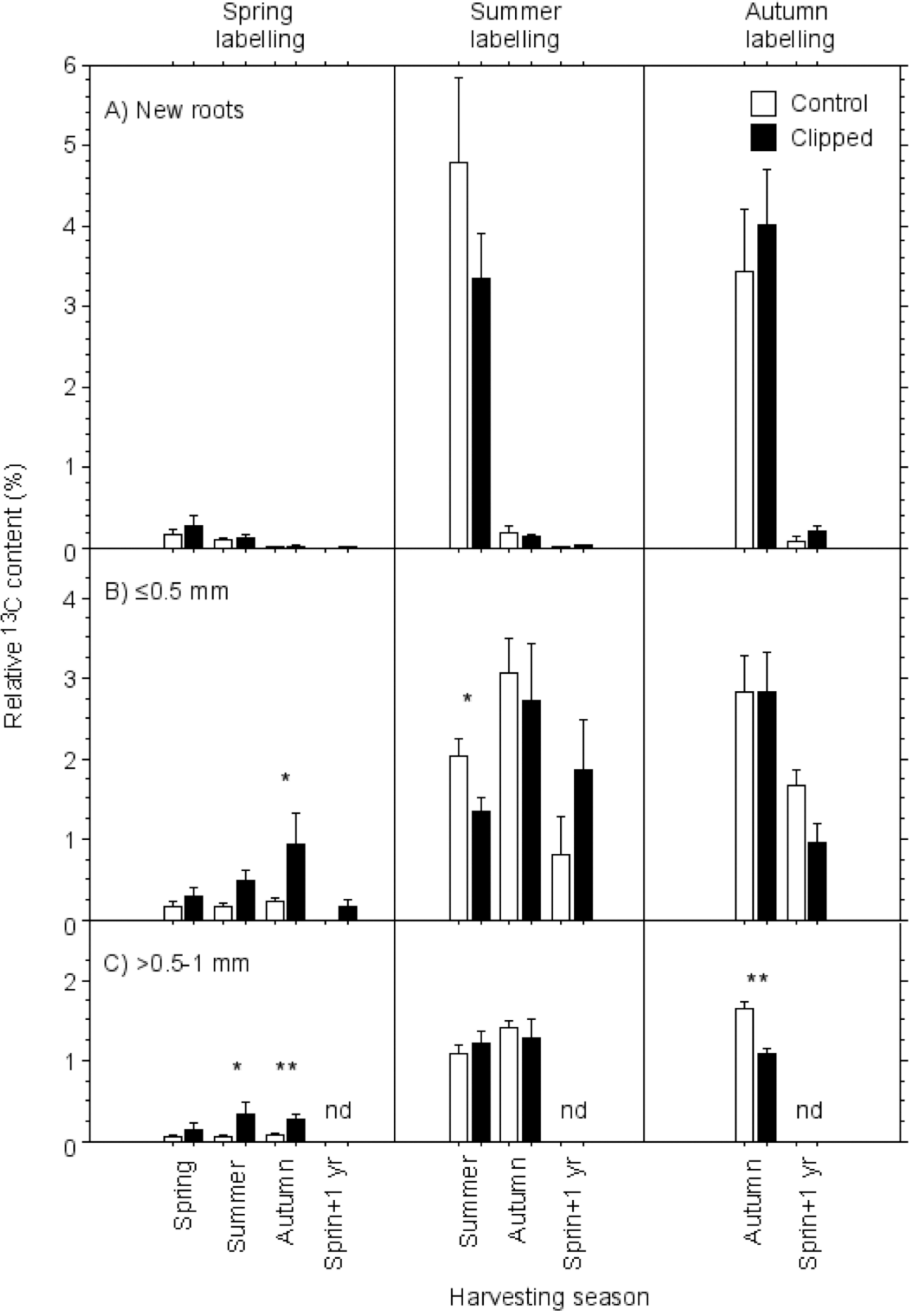
Means of relative ^13^C contents (%, ±SE; n = 4 for spring, summer, and autumn, n = 1–3 for spring+1 yr, see Table 1) recovered from bulk fine-roots of (A) new fine roots, (B) fine roots of class ≤0.5 mm, and (C) fine roots of class >0.5–1.0 mm for different seasons of ^13^CO_2_ pulse-labelling (spring, summer, autumn) and for different harvesting seasons. Relative ^13^C contents are expressed in percentage of the maximum needle signals. Significant differences between control and clipped trees are given with * (*P*<0.05) and ** (*P*<0.01).

Fine roots of the same diameter class of clipped and control trees had variable values in terms of ^13^C enrichment, expressed as relative ^13^C content (%), and the ^13^C enrichment differed strongly among seasons of labelling (Fig 2). Spring labelled trees had the lowest relative ^13^C contents (<1%) in the fine roots in comparison to summer and autumn labelled fine roots (Fig 2). Highest relative ^13^C contents (3–5%) were observed only in newly formed roots of the summer or the autumn harvests immediately after the ^13^CO_2_-labelling (Fig 2). Medium high ^13^C contents (1–3%) were recorded in fine roots of ≤0.5 mm and of >0.5–1.0 mm after the summer and the autumn labellings in all harvesting seasons including spring+1 yr (Fig 2).

Clipping the trees had significantly positive effects on the relative ^13^C content of the roots of the ≤0.5 mm and >0.5–1.0 mm root classes when the trees were labelled in spring (Fig 2), whereas clipping had significantly negative effects on the >0.5–1.0 mm root class when trees were labelled in autumn (Table 5). The harvesting season had a significantly positive effect on new roots and the ≤0.5 mm root class after summer labelling, with much higher relative ^13^C contents after the summer and autumn labelling (Table 5, Fig 2).

**Table 5:** Results of two-way ANOVA testing the effect of the clipping treatment (C) and the harvesting seasons (H) (spring, summer, autumn) on relative ^13^C contents (%) of three fine-root classes. Significant effects (*P*<0.05) are given in bold. (-: data not available).

Labelling season	Fine-root class	Clipping (C)			Harvesting season (H)			C x H		
		*df*	*F*	*P*	*df*	*F*	*P*	*df*	*F*	*P*
Spring	New roots	1	0.05	0.833	2	5.75	**0.012**	2	0.30	0.747
	≤0.5 mm	1	9.08	**0.008**	2	2.91	0.080	2	0.71	0.504
	>0.5–1.0 mm	1	14.16	**0.001**	2	3.78	**0.043**	2	0.09	0.914
Summer	New roots	1	1.00	0.337	1	222.34	**<0.001**	1	0.36	0.559
	≤0.5 mm	1	2.09	0.172	1	8.99	**0.010**	1	0.74	0.405
	>0.5–1.0 mm	1	0.05	0.836	1	1.00	0.338	1	0.79	0.393
Autumn	New roots	1	0.31	0.598	-	-	-	-	-	-
	≤0.5 mm	1	<0.01	0.994	-	-	-	-	-	-
	>0.5–1.0 mm	1	25.76	**0.002**	-	-	-	-	-	-

### Starch concentrations and recovered ^13^C label in starch

Highest starch concentrations (20–24 mg g^-1^) were found in newly grown fine roots in autumn (S2 Table). The clipping treatment did not significantly influence the starch concentrations in the new roots and the ≤0.5 mm fine root class, whereas the harvesting season influenced them in both root classes significantly (Table 6).

**Table 6:** Results of two-way ANOVA of the effect of the clipping treatment (C) and the harvesting seasons (H) (spring, summer, autumn) on the starch concentration and on the relative ^13^C content of the starch of two fine-root classes. Significant effects (*P*<0.05) are given in bold. (-: data not available).

Starch	Labelling season	Fine-root class	Clipping (C)			Harvesting season (H)			C x H		
			*df*	*F*	*P*	*df*	*F*	*P*	*df*	*F*	*P*
Concentration	-	New roots	1	0.67	0.418	2	25.05	**<0.001**	2	0.93	0.403
		≤0.5 mm	1	<0.01	0.973	2	6.01	**0.005**	2	0.32	0.731
Relative ^13^C content	Spring	New roots	1	2.81	0.116	2	11.87	**0.001**	2	0.48	0.632
		≤0.5 mm	1	7.13	**0.016**	2	2.39	0.121	2	0.76	0.483
	Summer	New roots	1	2.58	0.134	1	61.16	**<0.001**	1	0.84	0.377
		≤0.5 mm	1	0.09	0.771	1	0.67	0.434	1	2.00	0.188
	Autumn	New roots	1	3.00	0.134	-	-	**-**	-	-	-
		≤0.5 mm	1	0.19	0.668	-	-	-	-	-	-

**Fig 3:**
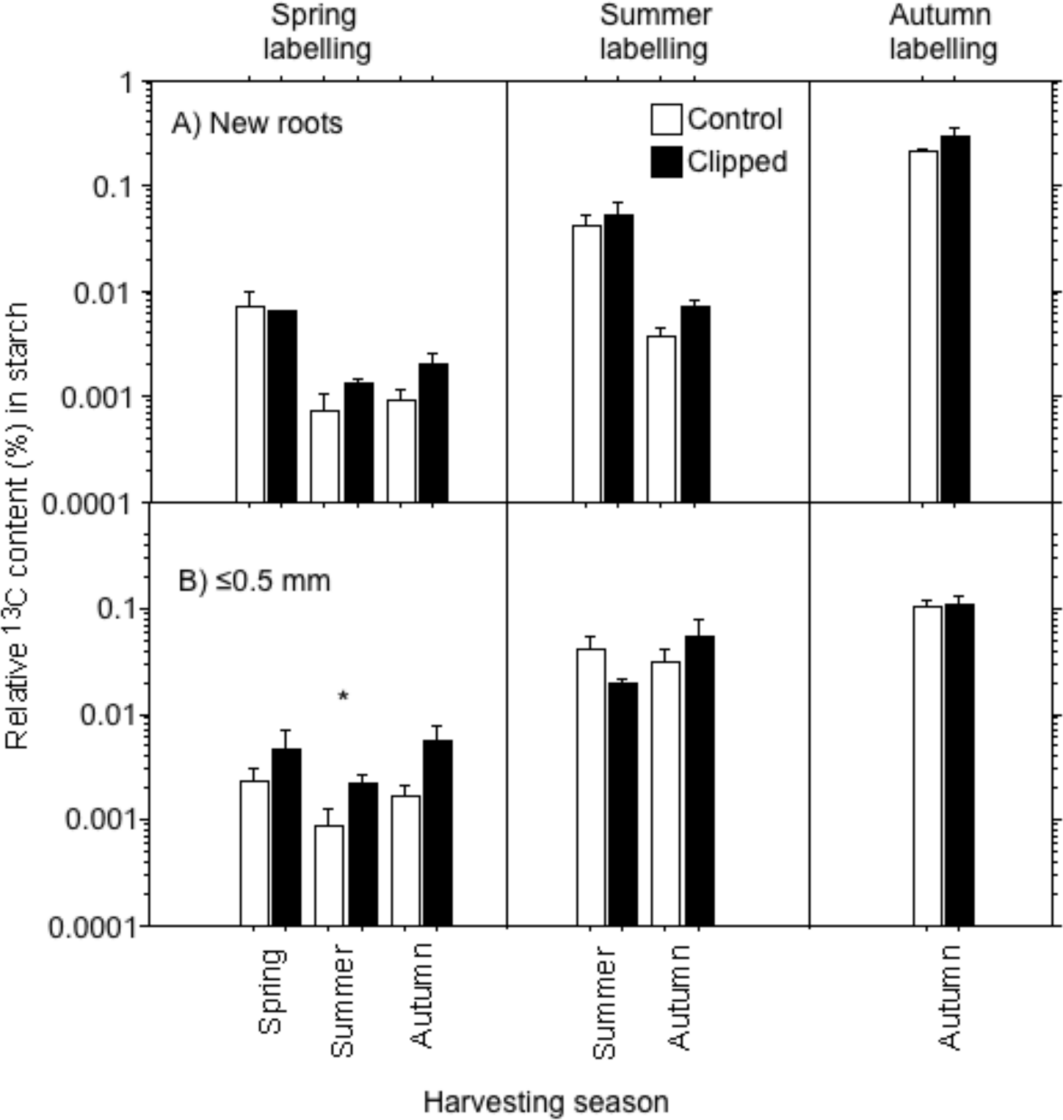
Means of relative ^13^C content (%, ± SE, n = 4) recovered from starch of (A) new fine roots and (B) of fine roots of class ≤0.5 mm for different seasons of ^13^CO_2_ pulse-labelling (spring, summer, autumn) and for different harvesting seasons (spring, summer, autumn) in controls and in clipped trees (logarithmic display). Relative ^13^C contents are expressed in percentage of the maximum needle signals. Significant differences between control and clipped trees are given with * (*P*<0.05).

Starch in the fine roots of spring ^13^CO_2_-labelled trees was only a little labelled with ^13^C, with mean relative ^13^C contents in starch below 0.01% (Fig 3). In contrast, plants labelled in summer allocated more label into the roots than those labelled in spring: mean relative ^13^C contents of new fine roots were up to 0.06% at the summer harvest (Fig 3). Highest starch ^13^C enrichments were found after the autumn labelling, with up to 0.3% in new roots and 0.1% in the ≤0.5 mm root class (Fig 3). Clipping had no significant effect on the relative ^13^C content of fine roots, except for the ≤0.5 mm root class after spring labelling (Table 6). In contrast, harvesting season had significant effects on ^13^C in new roots for the spring labelling as well as for the summer labelling (Table 6).

## Discussion

The aim of the present study was to investigate the effects of browsing, simulated through clipping, and of different seasons on the C allocation to the root systems of young *Abies alba* trees. The simulated browsing reduced the aboveground biomass immediately, since a large proportion of the branches have been cut off. The aboveground biomass was reduced mainly due to the clipped branches and due to the proportion of buds removed, and as a consequence, due to the lower number of new shoots produced in the clipped trees. This reduction is in accordance with other studies, e.g. the study of Hester et al. [9], reporting *Pinus sylvestris* trees having lost a similar amount of buds (58%) due to browsing, whereas the amount was lower in deciduous trees (43% in *Betula pendula* and 29% in *Sorbus aucuparia*, respectively). Nevertheless, we found compensatory growth of aboveground parts, e.g. shoots and needles, one year after clipping, as also reported by others [7,9,30]. More than half of the clipped trees built multiple stem forms, similar to results of Häsler et al. [10]. Clipping also affected belowground growth. Evidence of a short-term root growth due to the clipping of shoots was an up to 46% increased biomass in the fine-roots of the classes >0.5–1.0 mm, >1.0–1.5 mm, and >1.5–2.0 mm at spring and summer harvests. Babst et al. [31] as well found increased export of newly assimilated ^11^C (*t*_*1/2*_: 20.4 min) to roots in *Populus tremuloides* following jasmonic acid application (simulating a mechanical damage to leaves after feeding by insects). However, after a longer time period, as in our study after half a year or longer, simulated browsing usually results in a significantly reduced fine-root biomass of treated trees compared to control trees. Such belowground reductions were found as well in other tree species, such as *Quercus* spp., *Betula pubescens*, *Fagus sylvatica*, and *Abies alba*, after herbivore damage or defoliation [15,20,21,32,33]. Only a few studies, however, observed no belowground changes [18,34].

Starch concentrations in the fine roots of our study, in contrast to the biomass, did not show any changes due to the clipping treatment. Other studies made the same observation, that a 50% defoliation does not affect the amount of root starch [15,17,18,20,21,35]. Only a severe defoliation (100%), however, is able to diminish root starch significantly as shown by Piper et al. [18] with *Nothofagus* spp. or Jacquet et al. [17] with *Pinus pinaster*. Kosola et al. [35] additionally found that starch accumulation after defoliation occurred mainly in August, but not during the other measured seasons (early summer, late autumn). Overall, the above mentioned reports on root biomass and root starch showed that the severity of defoliation and the environmental circumstances (e.g. season of measurement) matter strongly.

Pulse-labelling with ^13^CO_2_ offers a straightforward method to track recently fixed photoassimilates from the shoots to the roots (e.g. [22,27,36,37]). Concerning our three hypotheses (1–3) postulated earlier, our data supported (1) and (2), but (3) only partly:

1. According to our first hypothesis, the seasons of the ^13^C-pulse-labelling and, thus, phenology had a strong effect on the allocation of the ^13^C-label from the shoot to the roots. Shoots labelled in spring did not supply the fine-roots with high amounts of the ^13^C-label, because the fine-roots had a relatively low ^13^C-content (<1%). Labelling in summer and autumn, however, revealed that relatively high amounts of the ^13^C-label (1–5%) were allocated to the fine roots. Thus, it can be assumed that photoassimilates from spring mainly remain in the shoots to support shoot growth, whereas photoassimilates from summer and autumn are allocated in larger portions to the fine roots (compare also [38]). Our findings correspond with reports by Ericsson et al. [39] and by Hansen et al. [40], using a ^14^CO_2_-label, who observed that spring assimilates were used mainly for aboveground growth, whereas autumn assimilates were allocated mainly to belowground parts as storage. Keel et al. [41] found similarly that ^13^C-labels from June were transported to the roots to a lesser amount compared to August. Moreover, Kuptz et al. [42] stated upon their labelling results that 'during spring, only negligible amounts of new photosynthates enter the transfer pool', but 'during early summer, new photosynthates enter the transfer pool, directly supplying growth and being further transported to roots and soils, and during late summer, new photosynthates enter both the transfer and storage pools, supplying both growth and maintenance' [42]. Our isotopic data fully support these findings of Kuptz et al. [42]. Subsequently, highest ^13^C-enrichments in the starch in the fine roots were recorded in our study in autumn after autumn labelling.
2. As proposed in our second hypothesis, the incorporation of the ^13^C-label into the roots was strongly dependent on the root type and the root diameter. Labelling in summer and autumn showed that relatively high amounts of the ^13^C-label (1–5%) were allocated to the fine roots, with 3–5% in newly formed fine roots, 1–3% in the ≤0.5 mm root class, and 1–1.5% in the >0.5–1.0 mm root class. Comparably, the absolute highest ^13^C-values in the starch were measured in the newly formed fine roots after the autumn labelling. Keel et al. [41], recording ^13^C-labels in starch likewise, observed highest values in the finest root class.
3. In accordance to our third hypothesis, clipping strongly influenced the amount of allocated ^13^C-label as well as the site of incorporation and the form of the incorporated ^13^C-label. Clipping had a significantly positive effects on the ^13^C-allocation to fine roots after spring labelling, with significantly higher relative ^13^C-contents observed in the ≤0.5 mm and the >0.5–1.0 mm fine-root classes of clipped trees than those of control trees. Similarly, significantly higher relative ^13^C-contents of starch were observed in the ≤0.5 mm fine-root class of clipped trees after spring labelling. After summer and autumn labelling, however, mainly no effects in the ^13^C-allocation to the fine roots were observed, assuming that clipping had not a strong effect on the ^13^C-allocation pattern.

Our study showed that clipping resulted in a reduction of the belowground biomass but not in an increase of the starch concentration in the roots. Thus, we conclude that no trade-off between growth and storage occurred. A trade-off would occur only if a C limitation would be present [43–45]. A sustained severe defoliation would lead to such a C limitation, with increased storage at the expense of growth enhancing the survival chance [45]. In general, tree growth can be limited by the availability of C within the plant (C limitation) or by the tree’s ability to use the available C it has because of e.g. nutrient shortage or environmental conditions (sink limitation) [44]. A growing body of literature suggests that current tree growth is sink limited under most conditions, with much of the evidence deducted from trees’ high amounts of nonstructural carbon (NSC) [44]. These conclusions rely on the assumption that C storage is passive and that NSC does not accumulate when growth is C limited. However, storage may be an active process, occurring at the expense of growth [44].

The processes behind the source-to-sink transport within plants are complex. Plants respond to herbivore feeding by increasing the production of the phytohormone jasmonic acid [46]. A systemic jasmonic acid induction of defense responses also occurs from roots to shoots and *vice versa*, thereby affecting the performance of herbivores. However, jasmonic acid also causes the re-allocation of primary metabolites between roots and shoots, and it controls several developmental processes such as root growth or senescence [46]. The molecular processes behind this source-to-sink transport most likely are phloem sucrose transport and invertases in sink organs, as the latter increase sucrose unloading by converting sucrose to hexoses [47,48].

In conclusion, our data imply that season of C assimilation and, thus, the seasonal phenology of trees is a main driver of C allocation from shoots to roots, and, as seen in our experiment, cleary more decisive than simulated browsing. However, within the fine-root system, the C allocation pattern varied greatly. Fine roots which are highly physiologically active, e.g. newly grown fine roots, received the highest amounts of newly assimilated C, but only after summer and autumn labelling. This indicates that newly formed fine roots are highly active only in the second half of the vegetation period in exploring the soil and storing C.

## Supporting Information

S1 Table Number of harvested trees. Number of harvested trees with the varying origins of new leader shoots for two clipping treatments and for four harvesting seasons. Treatments are 'Control' = unclipped trees, and 'Clipped' = clipped trees.
(DOCX)

S2 Table Means of starch concentrations. Means of starch concentrations (mg g^-1^ dry weight, ±SE) in new fine roots and in fine roots of class ≤0.5 mm for two clipping treatments and for three harvesting seasons. Treatments are 'Control' = unclipped trees, and 'Clipped' = clipped trees.
(DOCX)

## References

[1] Köstler JN, Brückner E, Bibelriether H. Die Wurzeln der Waldbäume: Untersuchungen zur Morphologie der Waldbäume in Mitteleuropa. Hamburg: Paul Parey; 1968.

[2] Robakowski P, Tomasz W, Samardakiewicz S, Kierzkowski D. Growth, photosynthesis, and needle structure of silver fir (*Abies alba* Mill.) seedlings under different canopies. For Ecol Manag. 2004;201: 211–227.

[3] Motta R. Impact of wild ungulates on forest regeneration and tree composition of mountain forests in the Western Italian Alps. For Ecol Manag. 1996;88: 93–98.

[4] Hester AJ, Bergman M, Iason GR, Moen J. Impacts of large herbivores on plant community structure and dynamics In: Danell K, Bergström R, Duncan P, Pastor J, editors. Large Herbivore Ecology, Ecosystem Dynamics and Conservation. Cambridge: Cambridge University Press;2006 pp. 97–141.

[5] Eiberle K. Ergebnisse einer Simulation des Wildverbisses durch den Triebschnitt. Schweiz Z Forstwes. 1975;126: 821–839.

[6] Gill RMA. A review of damage by mammals in North temperate forests: 1. Deer. Forestry. 1992;65: 145–169.

[7] Honkanen T, Haukioja E, Suomela J. Effects of simulated defoliation and debudding on needle and shoot growth in Scots pine (*Pinus sylvestris*): implications of plant source/sink relationships for plant-herbivore studies. Funct Ecol. 1994;8: 631–639.

[8] Duncan AJ, Hartley SE, Iason GR. The effect of previous browsing damage on the morphology and chemical composition of Sitka spruce (*Picea sitchensis*) saplings and on their subsequent susceptibility to browsing by red deer (*Cervus elaphus*). For Ecol Manag. 1998;103: 57–67.

[9] Hester AJ, Millard P, Baillie GJ, Wendler R. How does timing of browsing affect above- and below-ground growth of *Betula pendula*, *Pinus sylvestris* and *Sorbus aucuparia*? Oikos. 2004;105: 536–550.

[10] Häsler H, Senn J, Edwards PJ. Light-dependent growth responses of young *Abies alba* to simulated ungulate browsing. Funct Ecol. 2008;22: 48–57.

[11] Bardgett RD, Wardle DA. Herviore-mediated linkages between aboveground and belowground communities. Ecology. 2003;84: 2258–2268.

[12] Bezemer TM, van Dam NM. Linking aboveground and belowground interactions via induced plant defenses. Trends Ecol Evol. 2005;20: 617–624. 16701445 10.1016/j.tree.2005.08.006

[13] Väre H, Ohtonen R, Mikkola K. The effect and extent of heavy grazing by reindeer in oligotrophic pine heaths in northeastern Fennoscandia. Ecography. 1996;19: 245–253.

[14] Ruess RW, Hendrick RL, Bryant JP. Regulation of fine root dynamics by mammalian browsers in early successional Alaskan taiga forests. Ecology. 1998;79: 2706–2720.

[15] Palacio S, Hester AJ, Maestro M, Millard P. Browsed *Betula pubescens* trees are not carbon-limited. Funct Ecol. 2008;22: 808–815.

[16] Huttunen L, Saravesi K, Markkola A, Niemelä P. Do elevations in temperature, CO_2_, and nutrient availability modify belowground carbon gain and root morphology in artificially defoliated silver birch seedlings? Ecol Evol. 2013;3: 2783–2794. 10.1002/ece3.665 24101972 PMC3790529

[17] Jacquet JS, Bosc A, O’Grady A, Jactel H. Combined effects of defoliation and water stress on pine growth and non-structural carbohydrates. Tree Physiol. 2014;34: 367–376. 10.1093/treephys/tpu018 24736390

[18] Piper FI, Fajardo A. Foliar habit, tolerance to defoliation and their link to carbon and nitrogen storage. J Ecol. 2014;102: 1101–1111.

[19] Puri E, Hoch G, Körner C. Defoliation reduces growth but not carbon reserves in Mediterranean *Pinus pinaster* trees. Trees. 2015;29: 1187–1196.

[20] Wiley E, Huepenbecker S, Casper BB, Helliker BR. The effects of defoliation on carbon allocation: can carbon limitation reduce growth in favour of storage? Tree Physiol. 2013;33: 1216–1228. 10.1093/treephys/tpt093 24271085

[21] Palacio S, Paterson E, Sim A, Hester AJ, Millard P. Browsing affects intra-ring carbon allocation in species with contrasting wood anatomy. Tree Physiol. 2011;31: 150–159. 10.1093/treephys/tpq110 21388994

[22] Endrulat T, Saurer M, Buchmann N, Brunner I. Incorporation and remobilisation of ^13^C within the fine-root systems of individual *Abies alba* trees in a temperate coniferous stand. Tree Physiol. 2010;30: 1515–1527. 10.1093/treephys/tpq090 21076129

[23] Welch D, Staines BW, Scott D, French DD, Catt DC. Leader browsing by red and roe deer on young Sitka spruce trees in western Scotland. I. Damage rates and the influence of habitat factors. Forestry. 1991;64: 61–82.

[24] Kagawa A, Sugimoto A, Maximov TC. Seasonal course of translocation, storage and remobilization of ^13^C pulse-labeled photoassimilate in naturally growing *Larix gmelinii* saplings. New Phytol. 2006;171: 793–804. 16918550 10.1111/j.1469-8137.2006.01780.x

[25] Smith AM, Zeeman SC. Quantification of starch in plant tissues. Nature Protocols. 2006;1: 1342–1345. 17406420 10.1038/nprot.2006.232

[26] Regier N, Streb S, Cocozza C, Schaub M, Cherubini P, Zeeman SC, et al Drought tolerance of two black poplar (*Populus nigra* L.) clones: contribution of carbohydrates and oxidative stress defense. Plant Cell Environ. 2009;32: 1724–1736. 10.1111/j.1365-3040.2009.02030.x 19671097

[27] Göttlicher S, Knohl A, Wanek W, Buchmann N, Richter A. Short-term changes in carbon isotope composition of soluble carbohydrates and starch: from canopy leaves to the root system. Rapid Commun Mass Spectrom. 2006;20: 653–660. 16444688 10.1002/rcm.2352

[28] Philip LJ, Simard SW. Minimum pulses of stable and radioactive carbon isotopes to detect belowground carbon transfer between plants. Plant Soil. 2008;308: 23–35.

[29] Keel SG, Siegwolf RTW, Jäggi M, Körner C. Rapid mixing between old and new C pools in the canopy of mature forest trees. Plant Cell Environ. 2007;30: 963–972 17617824 10.1111/j.1365-3040.2007.01688.x

[30] Edenius L, Danell K, Bergström R. Impact of herbivory and competition on compensatory growth in woody plants: winter browsing by moose on Scots pine. Oikos. 1993;66: 286–292.

[31] Babst BA, Ferrieri RA, Gray DW, Lerdau M, Schlyer DJ, Schueller M, et al Jasmonic acid induces rapid changes in carbon transport and partitioning in *Populus*. New Phytol. 2005;167: 63–72. 15948830 10.1111/j.1469-8137.2005.01388.x

[32] Frost CJ, Hunter MD. Herbivore-induced shifts in carbon and nitrogen allocation in red oak seedlings. New Phytol. 2008;178: 835–845. 10.1111/j.1469-8137.2008.02420.x 18346100

[33] Ayres E, Heath J, Possell M, Black HIJ, Kersiens G, Bardgett RD. Three physiological responses to above-ground herbivory directly modify below-ground processes of soil carbon and nitrogen cycling. Ecol Lett. 2004;7: 469–479.

[34] Kosola KR, Dickmann DI, Paul EA, Parry D. Repeated insect defoliation effects on growth, nitrogen acquisition, carbohydrates, and root demography of poplar. Oecologia. 2001;129: 65–74. 28547069 10.1007/s004420100694

[35] Kosola KR, Dickmann DI, Parry D. Carbohydrates in individual poplar fine roots: effects of root age and defoliation. Tree Physiol. 2002;22: 741–746. 12091156 10.1093/treephys/22.10.741

[36] Blessing CH, Werner RA, Siegwolf R, Buchmann N. Allocation dynamics of recently fixed carbon in beech saplings in response to increased temperatures and drought. Tree Physiol. 2015;35: 585–598. 10.1093/treephys/tpv024 25877767

[37] Epron D, Bahn M, Derrien D, Lattanzi FA, Pumpanen J, Gessler A, et al Pulse-labelling trees to study carbon allocation dynamics: a review of methods, current knowledge and future prospects. Tree Physiol. 2012;32: 776–798. 10.1093/treephys/tps057 22700544

[38] Abramoff RZ, Finzi AC. Are above- and below-ground phenology in sync? New Phytol. 2015;205: 1054–1061. 25729805 10.1111/nph.13111

[39] Ericsson T, Rytter L, Vapaavuori E. Physiology of carbon allocation in trees. Biomass Bioenerg. 1996;11: 115–127.

[40] Hansen J, Vogg G, Beck E. Assimilation, allocation and utilization of carbon by 3-year-old Scots pine (*Pinus sylvestris* L.) trees during winter and early spring. Trees. 1996;11: 83–90.

[41] Keel SG, Campbell CD, Högberg MN, Richter A, Wild B, Zhou X, et al Allocation of carbon to fine root compounds and their residence times in a boreal forest depend on root size class and season. New Phytol. 2012;194: 972–981. 10.1111/j.1469-8137.2012.04120.x 22452424

[42] Kuptz D, Fleischmann F, Matyssek R, Grams TEE. Seasonal pat- terns of carbon allocation to respiratory pools in 60-yr-old deciduous (*Fagus sylvatica*) and evergreen (*Picea abies*) trees assessed via whole-tree stable carbon isotope labeling. New Phytol. 2011;191: 160–172. 10.1111/j.1469-8137.2011.03676.x 21395596

[43] Millar P, Way DA. Tree competition and defense against herbivores: currency matters when counting the cost. Tree Physiol. 2011;31: 579–581. 10.1093/treephys/tpr053 21778292

[44] Wiley E, Helliker B. A re-evaluation of carbon storage in trees lends greater support for carbon limitation to growth. New Phytol. 2012;195: 285–289. 10.1111/j.1469-8137.2012.04180.x 22568553

[45] Palacio S, Hoch G, Sala A, Körner C, Millard P. Does carbon storage limit tree growth? New Phytol. 2014;201: 1096–1100. 10.1111/nph.12602 24172023

[46] Tytgat TOG, Verhoeven KJF, Jansen JJ, Raaijmakers CE, Bakx-Schotman T, Lauren M, et al Plants know where it hurts: Root and shoot jasmonic acid induction elicit differential responses in *Brassica oleracea*. PLoS ONE. 2013;8: e65502 10.1371/journal.pone.0065502 23776489 PMC3679124

[47] Klopotek Y, Franken P, Klaering HP, Fischer K, Hause B, Hajirezaei MR, et al A higher sink competitiveness of the rooting zone and invertases are involved in dark stimulation of adventitious root formation in *Petunia* hybrida cuttings. Plant Sci. 2016;243: 10–22. 10.1016/j.plantsci.2015.11.001 26795147

[48] Lemoine R, La Camera S, Atanassova R, Dédaldéchamp F, Allario T, Pourtau N, et al Source-to-sink transport of sugar and regulation by environmental factors. Front Plant Sci. 2013;4: 272 10.3389/fpls.2013.00272 23898339 PMC3721551

