# Supplementary material for: Carbon Allocation into Different Fine-Root Classes of Young Abies alba Trees Is Affected More by Phenology than by Simulated Browsing
Source: PLoS One. 2016 Apr 28;11(4):e0154687. doi: 10.1371/journal.pone.0154687 (PMC4849635; doi:10.1371/journal.pone.0154687)
Supplement: S1 Table — Number of harvested trees with the varying origins of new leader shoots for two clipping treatments and for four harvesting seasons. Treatments are 'Control' = unclipped trees, and 'Clipped' = clipped trees. (DOCX) [file pone.0154687.s001.docx]

**S1 Table**. Number of harvested trees with the varying origins of new leader shoots for two clipping treatments and four harvesting seasons. Treatments are 'Control' = unclipped trees, and 'Clipped' = clipped trees.

| Treatment | Harvesting season | Origin of the new leader shoots | | | | Sum of trees |
| --- | --- | --- | --- | --- | --- | --- |
|  |  | From apical buds | From interwhorl buds | From secondary whorl buds | Without new leader shoots |  |
| Control | Spring | 4 | 0 | 0 | 1 | 5 |
|  | Summer | 9 | 0 | 0 | 0 | 9 |
|  | Autumn | 13 | 0 | 0 | 0 | 13 |
|  | Spring+1 yr | 6 | 0 | 0 | 0 | 6 |
|  | Sum of trees | 32 | 0 | 0 | 1 | 33 |
| Clipped | Spring | 0 | 1 | 3 | 1 | 5 |
|  | Summer | 0 | 6 | 3 | 0 | 9 |
|  | Autumn | 0 | 4 | 8 | 1 | 13 |
|  | Spring+1 yr | 0 | 3 | 2 | 3 | 8 |
|  | Sum of trees | 0 | 14 | 16 | 5 | 35 |
