# Supplementary material for: Carbon Allocation into Different Fine-Root Classes of Young Abies alba Trees Is Affected More by Phenology than by Simulated Browsing
Source: PLoS One. 2016 Apr 28;11(4):e0154687. doi: 10.1371/journal.pone.0154687 (PMC4849635; doi:10.1371/journal.pone.0154687)
Supplement: S2 Table — Means of starch concentrations (mg g-1 dry weight, ±SE) in new fine roots and in fine roots of class ≤0.5 mm for two clipping treatments and for three harvesting seasons. Treatments are 'Control' = unclipped trees, and 'Clipped' = clipped trees. (DOCX) [file pone.0154687.s002.docx]

**S2 Table**. Means of starch concentrations (mg g^-1^ dry weight, ±SE) in new fine roots and in fine roots of class ≤0.5 mm for two clipping treatments and for three harvesting seasons. Treatments are 'Control' = unclipped trees, and 'Clipped' = clipped trees.

| Harvesting season | New roots | | ≤0.5 mm root class | |
| --- | --- | --- | --- | --- |
|  | Control | Clipped | Control | Clipped |
| Spring | 11.3±1.9 | 10.0±4.0 | 19.3±4.9 | 19.3±6.8 |
| Summer | 9.5±1.1 | 12.6±1.2 | 10.0±1.6 | 9.9±1.0 |
| Autumn | 19.9±1.8 | 24.6±2.0 | 15.5±2.1 | 16.1±1.1 |
